# Reports from the frontline: health workers describe COVID-19 risks and fears in five cities in Brazil

**DOI:** 10.1186/s12913-023-09118-y

**Published:** 2023-03-22

**Authors:** Carl Kendall, Ana Ecilda Lima Ellery, Nivaldo Carneiro Junior, Rosane da Silva Santana, Luciane Nascimento Cruz, Mírian Cohen, Marto Leal, Luana Nepomuceno Gondim Costa Lima, Maria Amélia de Sousa Mascena Veras, Maria de Fátima Pessoa Militão de Albuquerque, Karla Valéria Batista Lima, Celina Maria Turchi Martelli, Ligia Regina Franco Sansigolo Kerr

**Affiliations:** 1 School of Public Health and Tropical Medicine, Tulane University, New Orleans, LA USA; 2 Federal University of Ceará, Programa de Pós-Graduação Em Saúde Pública, Fortaleza, Brazil; 3 Faculdade de Ciências Médicas da Santa Casa de São Paulo (FCMSCSP), São Paulo, Brazil; 4 Federal University of Rio Grande do Sul, Porto Alegre, Brazil; 5 Instituto Evandro Chagas, Belem, PA Brazil; 6 Brazil, Oswaldo Cruz Foundation - Pernambuco (FIOCUZ-PE), Recife, Brazil

**Keywords:** COVID-19, Brazil, Health workers, Qualitative research, Rapid ethnographic assessment

## Abstract

**Background:**

Health providers are under unprecedented pressures to perform in the COVID-19 health crisis and under unprecedented risks. We initiated a large mixed-method survey of health professionals in five large metropolitan areas in Brazil to document the risks and needs of health professionals. To initiate the study, we conducted formative research.

**Methods:**

We conducted 77 open-ended semi-structured interviews online in a convenience sample of physicians, nurses, nurse technicians, and physiotherapists in Belem, Fortaleza, Porto Alegre, Recife, and São Paulo, Brazil. Design, data collection, and analysis were informed by Rapid Ethnographic Analysis (REA).

**Results:**

Responses are organized into three themes that emerged in the interviews: the lack of preparation – both locally and nationally—for the pandemic and its effects on staffing and training; the overlap of personal, family, and professional risk and consequences; and inadequately addressed anxiety and suffering among health staff.

**Conclusions:**

Our respondents were unprepared for the epidemic, especially the institutional sequelae and psychological cost. These consequences were exacerbated by both lack of leadership and sweeping changes undercutting the Brazilian health system noted by almost all participants.

## Introduction

Compared to the general population during the pandemic of SARS-CoV-2 (COVID-19), health professionals are a highly vulnerable group at the center of transmission. It is estimated that approximately 14% of global cases of COVID-19 reported by October 2020 were among health professionals [1]. In Brazil, almost 40,000 cases of COVID-19 were confirmed among health professionals by the beginning of 2021 (BRASIL, 2021). The most affected professional groups in the country were nursing technicians (29.8%), nurses (17.1%), and physicians (11.9%) [2].

The high incidence of COVID-19 among health professionals is unsurprising, given that they care for infected patients and, consequently, are regularly exposed to infection. In addition, the Brazilian Federal and some state governments have been negligent in their response to the epidemic, avoiding evidence and science-based coordination and planning, failing to use financial resources provided for COVID-19, such as for vaccine purchase and personal protective equipment (PPE), and in general creating precarious working conditions in hospitals and clinics that made COVID-19 a much more serious epidemic in Brazil than it had to be.

In response to the COVID-19 pandemic, most health professionals were forced to change their work routines, increasing their workloads or moving from their original jobs—most often not an ICU or emergency service—to treating patients with COVID. Such changes, combined with poor working conditions, exhausting hours, insufficient training, and lack of personal protective equipment made the work environment a hostile one, directly affecting the performance and health of the team in the fight against COVID-19 [3, 4].

These factors also contributed to an increased risk of developing mental disorders during the COVID-19 pandemic. Symptoms of anxiety, depression, insomnia, problematic alcohol use, fear of becoming infected and/or transmitting the virus to friends and family, and psychosomatic symptoms increased among health professionals [5].

With this as background, the objectives of this study were to prepare for the quantitative component of “Risk assessment of health professionals who care for people with COVID-19 (CNPq 402,403/2020–7), PI Dr. Ligia Kerr. This study was meant to explore the impact of COVID-19 on all the health professionals who came in contact with COVID-19 in performance of their jobs across the different regions of Brazil. This formative research thus explored -through a qualitative lens—the effects of COVID-19 on different health professionals in 5 regions of the country. This paper reports those findings.

## Methods

The research reported here was conducted between April and September 2020, with healthcare professionals treating suspected or confirmed cases of COVID-19 in the metropolitan areas of: Belem (BEL), Fortaleza (FOR), Porto Alegre (POA), Recife (REC) and São Paulo (SP).

A convenience sample was selected using recommendations from known contacts in each city. The health professionals included were physicians (P), nurses (N), and nursing technicians (NT).

We applied Rapid Ethnographic Assessment (REA) [6]. REA is a collection of tools for conducting qualitative research derived primarily from applied anthropology. REA encompasses many observational and interview methods, but primarily is characterized by a relatively large number of semi-structured and open-ended interviews conducted over a brief period of time, often 3 months. Since research objectives drive method, and speed is important, analysis may consist of structured coding with multiple investigators, as in the grounded theory approach, but may also use narrative, ethnographic and other strategies for analysis. We used semi-structured open-ended research guides with P, N, and NT in each of the five cities city to explore the logistics of conducting the larger study as well as to collect a first-hand qualitative account of the issues facing our respondents. Data collected included age, profession, employment history, social network, and topics related to COVID-19 (risk perception and comorbidities, training received for COVID-19, mental health, and burnout). The interviews were conducted online by project staff, project directors in each city, and public health graduate students following training, piloting, and review of the research guide. Contacts who agreed to participate were read the consent form and sent an electronic version that they approved and returned to the study. The interview proceeded then or an appointment was made to follow up. Interviews were recorded and transcribed with explicit permission of the respondent. Analysis proceeded for the dual goals of providing information for the design and conduct of the quantitative study and to develop a descriptive paper of the findings. Analysis used targeted reading to summarize main results and a priori themes, as well as to identify emergent themes, a topic of much discussion. As a first approach to characterizing the data, spreadsheet matrices were developed. Each interview was listed by row and several versions of column labels identifying sociodemographic and professional characteristics, availability of PPE, personal experience with COVID-19 and other experiences were explored. Important paraphrases of data and illustrative quotes were identified. From this review, AELE took the lead in developing an early long draft of the paper in Portuguese. This was shared and discussed by the author team. Over several revisions consensus was developed, priority topics identified and the paper reduced to its current size.

The research was approved by the Research Ethics Committee of FIOCRUZ Instituto Aggeu Magalhães (authorization no.^.^ 4,021,099). All procedures were performed in accordance with relevant guidelines. For reasons of confidentiality, where cited, respondents are identified only by the professional category, age, and metropolitan region where they work.

## Results

A total of 77 health professionals were interviewed: 40.2% physicians, 27.3% nurses, 24.7% nursing technicians, and 7.7% physical therapists. Women constituted the majority of the sample (57.1%); 66.1% were between 31 and 50 years of age; 96.1% worked in the state capitals; 64.9% worked in public hospitals; 53.2% reported having been infected with COVID-19 and 25.9% reported complications from the disease (Table 1).

**Table 1:** Characterization of health professionals' professionals. *N* = 77

Variables	n	%
Age group		
21 to 30	15	19,4
31 to 40	25	32,4
41 to 50	26	33,7
51 to 60	11	14,2
Sex		
Female	44	57,1
Male	33	42,9
Professional category:		
Physician	31	40,2
Nurse	21	27,3
Nurse Technician	19	24,7
Physiotherapist	6	7,8
Job location		
Capital city	74	96,1
Metropolitan region	9	11,7
Workplace^a^		
Basic Health Unit	6	7,8
Urgent care clinic	4	5,2
Public Hospital	50	64,9
Private Hospital	19	24,7
Emergency ambulance services	5	6,5
Doctor’s office/ home visits	8	10,3
Other	12	15,6
Number of places worked		
1	35	45,4
2	29	37,6
3	8	10,3
4	5	5,1
5 or more	2	2,5
Re-assigned to COVID-19 services		
Yes	18	23,3
No	17	22,1
No response	42	54,6
Comorbidities^a^		
Diabetes	6	7,8
Hypertension	14	18,1
Overweight/Obese	12	15,5
Coronary Disease	2	2,6
Kidney Disease	1	1,3
Asthma	4	5,2
None	49	63,6
Previously infected with Sars-Cov-2		
Sim	41	53,2
Não	36	46,7
Complications of COVID-19^a^		
Physical	20	25,9
Neurological	1	1,3
Psychological	6	7,8
None	19	24,6
No Response	38	49,3

^a^ More than one workplace or more than one comorbidity or more than one complication of COVID-19

Three major themes emerged in analysis: the acceleration of the pandemic and the shock and lack of preparedness of health services; overlapping risk inside and outside the workplace; and anxiety and suffering among health workers.

### Pandemic acceleration and the shock to services

According to our respondents, even though the world and Brazil have experienced other contagious diseases, nothing in the last 100 years could be compared to the COVID-19 pandemic. The pandemic generated a sudden change in both work and family routines for workers who had to adapt quickly to this new threat full of uncertainties.

The emergency presented by the epidemic, the rapid transmission and the sheer lethality of COVID-19 meant that modifications to the physical infrastructure and the training of work teams was incomplete. To some extent, “war strategies” were used, with the opening of field hospitals, participation of newly graduated staff, early graduation and recruitment of student health professionals, and assignment of staff with no training or experience in clinical care in infectious disease settings, including assigning administrative health staff to care services. The response of the health system was improvisatory and amateurish, with inadequate physical structure, lack of personal protective equipment (PPE), diagnostic kits and medications, routine exposure to infection, little formal training about the disease, and procedures and uncertain poorly implemented requirements for clinician isolation.

*It was a sudden, radical change, due to the fear of the unknown (...). It was hopeless (...) it was a feeling that you knew you were on the precipice and at any time you could die or fall. It was a very strange feeling (N, 48y, POA).*

*The worst moment was April 2020 (...). All of a sudden, in a week, it was chaos! Nobody believed this was happening. Then a lot of people died. We didn’t know how to treat [them]. (P, 26y, FOR).*

As a new disease, there was limited scientific evidence on how to properly treat patients. This triggered a feeling of impotence, and the fear of not responding adequately to the patients’ needs.

*Not having a theoretical base to support decisions ... Seeing such serious cases made us extremely insecure. Colleagues abusing medication without any scientific evidence… (P, 44y, BEL).*

Regarding preparation for COVID-19, most professionals reported receiving some type of training, mainly on donning and removing PPE. However, most respondents considered this training inadequate, referring to the absence of protocols for work routines and biosecurity, and their subsequent need to rely on colleagues and varied sources for self-education.

Brazil’s federal government’s negligence in confronting the pandemic, which engendered a sense of chaos, had broad repercussions on health and health care workers generated great anger, mildly and carefully expressed here:

I felt politicians’ neglect of human beings. The president dismissed the disease. It was a disappointment (P, 56y, SP).

This sentiment was voiced by almost all participants, often in stronger language. Other health and local authorities came in for criticism as well, but participants often mentioned unproven medications being promoted by the President, decisions about vaccine purchases that delayed Brazil’s vaccine program and funding cuts that reduced clinic staff.

### Overlapping risks inside and outside the workplace

Staff pointed out many points of vulnerability in work routines that increased their risk of infection. Respondents highlighted the insufficiency and poor quality of the PPE, their restricted access to testing, the inadequacy of their physical infrastructure, the insufficiency of their training, and sheer physical exhaustion:

*The work overload, the physical exhaustion, the discouragement, the situation in which we don and remove [PPE], all of this influences us to let down our guard and infect ourselves (N, 37y, REC).*

Discomfort using PPE was a recurrent complaint. The limited availability, often restricted to two sets for the long hours on duty, were not enough. Thus, health workers could not remove their gowns and equipment as often as necessary to cool down, hydrate or to use the toilet. Some female staff complained of urinary infections. Masks were also too rare for correct use, for example one nurse reported that N95 masks were reused for seven days, even under extremely hot conditions in her clinic.

Regarding procedures and the risk of infection, any proximity to patients, but especially physical exam and blood draws were considered a time of great risk:

*The physical exam is very stressful. Collecting tests (…) any situation where you need to be in a closed environment with the patient is stressful. When you enter the hospital, the emergency, you already feel a tension, a feeling of insecurity. (P, 43y, POA).*

In the absence of information, there was no uniformity among professionals about which clinical services would be at higher risk. Some cited the Emergency or the ICU, others, primary care or home visits. In general, each professional category considered the service or workplace they work in as the one with the greatest risk:

*Patients are using non-invasive ventilation, which generates aerosol. So I believe that we have high risk, high exposure, but we also receive all the necessary equipment. (N, 48y, POA)*

*We go to people's homes and assist the sick person... [this means] we often serve needy populations, where the demographic density within the household is large (...) This is a type of risk that we are involved in (NT, 33y, STR).*

While hospitals received the most ill patients, they were also better equipped. However, insufficient beds in main hospital wards meant that patients have a prolonged stay in ambulances where visiting and more peripheral staff have an increased risk of infection.

The risk of infection is not only present in patient care environments, but also in lavatories and in cafeterias where masks are removed:

*Infection occurs a lot among colleagues, touching door handles, using the same bathrooms, and sleeping in the same room…For meals, the dining area is very small, and they take off their masks. (NT, 49y, REC).*

### Anxiety and suffering among health professionals

Maintaining distance, especially emotionally, is critical to the rationalist and mechanistic view of biomedicine, and to the distance required to treat patients. With high levels of disease and death among health professionals COVID-19 challenged this distance, generating great fear, anxiety, and sadness among health care workers. Often these feelings were repressed because they were seen as signs of weakness and incompetence. When fear overwhelms many practioners, remarked several respondents, they stop practicing.

In the absence of even basic definitive knowledge, the rationalist view of health is, in itself, a generator of suffering, especially when there is no one listening to their needs and suffering at work. Left alone, with no systemic response in place, there is a greater risk of professionals seeking refuge in alcohol, in smoking, or even in the abuse of psychotropic drugs as strategies to cope with stress and low vitality.

Several days we had to choose between an older patient and a 30-year-old. There were days when I came home sad and drank more, because I couldn't stand it. (P, 26y, STR).

I went back to smoking (I was quitting). (…) Other colleagues were in the same situation. (P, 50y, SP).

Another common symptom was sleeplessness:

*When I went to sleep at home, I kept thinking: My God, am I doing it wrong? Should I be isolated? I didn't even sleep well. (...) I kept reviewing the cases in my head. (P, 41y, BEL).*

The fear of infecting family members was recurrent, being one of the main causes of emotional distress. Many health professionals left their homes, especially multigenerational households (which are not uncommon) and rented apartments or stayed in hotels, which led to greater anxiety and suffering.

*When I contracted COVID-19, I decided not to go to my parents' house, to protect them. It was April 2020. The pandemic was raging. (…) I decided to go to a hotel (…) At the hotel, we couldn't leave the room. (…) It was bad to be isolated. I spent 30 days there. You look crazy (…) [ I] cannot study, read, or exercise (P, 26y, STR).*

The workload to which the teams of professionals were submitted greatly contributed to problems such as physical exhaustion, with frequent complaints of burnout.

*I went into burnout diagnosed by a psychiatrist and I'm taking medication. If I talk a lot about it, I start to cry… (P, 33y, SP).*

Dealing with deaths, many of them preventable, were moments of intense suffering, with projections of their own vulnerability and limitations:

*The hardest moment was not being able to provide assistance to patients. (...). In a fateful shift, I started the shift with 7 dead. When I received the containers [for bodies] they were all over. No one else could fit. (P, 26y, FOR)*

In this stressful environment with an overwhelming workload, conflicts between workers are natural. Management underscored how challenging it was to deal with recurrent conflicts in the work team. This requires an ability, especially from leaders, to manage differences and personal boundaries.

*There is the issue of conflicts in the team. Knowing who you can count on, who is the most difficult, who is most afraid; in respecting people's limits more, because each one has a limit of what they can achieve. There are people who became very psychologically ill (N, 37y, POA).*

On the other hand, staff encountered barriers in communicating with managers and a fear of reprisal. Many health professionals are contracted, temporary or part-time, with precarious links to the system:

*Professionals are afraid to complain and come to complain to me [as co-workers, not management]. They cry in anguish, afraid of getting sick, afraid of transmitting the virus to their children, because they see and know how high the lethality is... I feel very lonely. (N, 48y, REC)*

Our participants were emphatic about the benefits of participating in the study, that it provided an opportunity to express silent and silenced needs, giving visibility to the numerous shortages experienced, and to hidden feelings.

*I think it's very good, because in this research we are going to externalize what we experience, how we behave in an unknown situation, our fears (...). The professional will have his voice. (N, 44y, BEL).*

Taking care of the caregiver, in this case, the health professionals, was a need that was abundantly expressed. Paraphrasing several participants, taking care of professionals is not only providing PPE and staff. Among topics mentioned were assistance with additional expenses such as purchase of PPE, assistance for housing during isolation, and social and psychological counseling.

## Discussion

The study showed that healthcare professionals working on the front lines in the care of patients with COVID-19 were profoundly affected by the pandemic in multiple ways. Rather than lead, Brazil’s federal government attempted to downplay the epidemic, restricting purchase of vaccines, adopting marginal and unproven therapies such as hydrochloroquine and ivermectin, fighting mask-wearing, distancing, and lockdowns, and attributing the call for a consistent science-based response to the epidemic a failure of masculine toughness. These tragic absurdities left local authorities to respond to the pandemic on an ad hoc basis and led to higher morbidity and mortality in the general population and among health professionals. Providers were left to address the disaster that followed, facing a public implicitly or explicitly blaming them for the consequences of the federal government’s ineptitude, i.e., lack of staff, beds, equipment and supplies and rising deaths. The challenge to health authority explicit in the political response in Brazil only added to the burden of health workers. How to resolve the contradiction of practicing science-based medicine when privileged parts of society are broadly challenging science as a basis for prevention and treatment, led by national leaders?

Many of these consequences have been documented. Contact with COVID-19 patients forced providers to avoid their own families and communities, leading to social isolation. The fear of becoming infected was constant. Add that to work overload, shortages of infrastructure and PPE, the loss of colleagues and patients, and great gaps in understanding a disease with high morbidity and mortality created a challenge that is unprecedented in modern times [7].

Studies in China emphasize the occurrence of intense psychological suffering, with the development of symptoms of insomnia, anxiety, and depression [8, 9]. In Brazil, the situation is similar. Research indicates that about 50% of health professionals have symptoms indicative of depression and/or anxiety [5]. Interestingly, few respondents mentioned any psychological consequences of infection with SARS-CoV-2, narrowly focusing their responses to the question to one's own experience of the disease (Table 1). Direct sequelae of infection were not considered. Training is certainly required to provide tools for health professionals to cope.

These factors are aggravated by the social and political context of Brazil, where long-established public policies to support science and education were being neglected, with drastic budget cuts (up to 50%) for public health, science, and technology, unlike China or other advancing countries. These trends, which preceded the epidemic, came at a time when global comparisons were being used by the Federal government to disparage Brazil’s workforce in general [10]. Ultimately, this personal crisis for health staff during the pandemic became an opportunity for the previous Federal government and its neoliberal policies to shrink the public sector. Such a choice, in a country as poor and unequal as Brazil will lead to increasing poverty and health disparities. How the performance of the health sector under COVID-19 is interpreted and used in political and economic discourse is yet to be determined. If we have learned anything from the pandemic in Brazil and the United States, it is how extremely political health and healthcare is. The consequences of this mismanagement are inscribed on the lives and careers of thousands of health professionals who were at the forefront of COVID-19 care during the pandemic.

## Data Availability

The datasets generated and/or analyzed uring the current study are not yet publicly available because the project is ongoing and are still being analyzed by the project team. This data is available from the corresponding author on reasonable request for scientific purposes.
